# Management of acutely decompensated chronic thromboembolic pulmonary hypertension in late pregnancy: a case report

**DOI:** 10.1186/s12884-019-2545-7

**Published:** 2019-10-21

**Authors:** Luca Valko, Gyorgyi Csosza, Akos Merei, Diana Muhl, Reka Faludi, Kristof Karlocai, Andras Lorx, Janos Gal

**Affiliations:** 1 Department of Anesthesiology and Intensive Therapy, Semmelweis University, Ulloi ut 78/B, Budapest, 1082 Hungary; 2 Department of Pulmonology, Semmelweis University, Dios arok u. 1, Budapest, 1125 Hungary; 3 Department of Anesthesiology and Intensive Therapy, Medical School, University of Pecs, Ifjusag u. 13, Pecs, 7624 Hungary; 4 Heart Institute, Medical School, University of Pecs, Ifjusag u. 13, Pecs, 7624 Hungary

**Keywords:** Maternal morbidity, Late pregnancy, Pulmonary hypertension, Chronic thromboembolic pulmonary hypertension, Right heart failure

## Abstract

**Background:**

Pregnancy in patients with pulmonary hypertension is associated with increased risk of maternal and fetal death. Physiological changes during pregnancy, labor and the postpartum period may all lead to acute decompensation of chronic right heart failure with rapid progression to circulatory collapse. As such, guidelines discourage planned pregnancies in women suffering from pulmonary hypertension. There are, however, rare cases of pulmonary hypertension which have previously been undiagnosed and only become apparent during late stage pregnancy. These individuals require close monitoring and multidisciplinary management.

**Case presentation:**

We describe the case of a 34-year-old female who presented with acute decompensation of previously undiagnosed pulmonary hypertension during the 30th week of her second pregnancy. Echocardiography and CT scan confirmed severe pulmonary hypertension and right heart failure with no new thromboembolic component. Following stabilization of cardiorespiratory parameters with high FiO_2_ noninvasive ventilation, intravenous epoprostenol and levosimendan treatment, Cesarean section was performed under epidural anesthesia. Close monitoring was continued in the postoperative period and cardiovascular parameters were managed with ongoing inotropic and escalating vasodilator therapy. The findings were consistent with chronic thromboembolic pulmonary hypertension. Persistent hypoxia was found to be a result of right bronchial obstruction caused by blood clots, which resolved with bronchoscopic intervention. Ongoing postpartum management resulted in improved cardiovascular parameters and oxygenation. Epoprostenol treatment was successfully converted to subcutaneous treprostinil therapy and the patient was discharged home to care for her healthy baby girl. Optimal timing of pulmonary endarterectomy will be chosen based upon functional status and patient preference.

**Conclusions:**

The case described is the first published report of previously undiagnosed pulmonary hypertension presenting with acute right heart failure in late pregnancy successfully managed by pharmacological therapy, noninvasive ventilation and a Cesarean performed under epidural anesthesia. The case illustrates that despite the challenges, acutely discovered right heart failure can be successfully managed with a comprehensive multidisciplinary treatment plan.

## Background

Pre-pregnancy comorbidities are a known associated risk factor for increasing severe maternal morbidity and mortality affecting even high-income countries [1]. Pulmonary hypertension, a multietiological disease leading to chronic right heart failure, is such a possible comorbidity [2]. Acute decompensation of the condition can lead to rapid progression, multiorgan failure and cardiovascular collapse, with an extremely high mortality rate of 14–100% [3]. The most severe forms of the disease, including idiopathic pulmonary arterial hypertension (IPAH) and chronic thromboembolic pulmonary hypertension (CTEPH), often affect young females [2, 4] and it is well known that pregnancy in these conditions carries significant additional risk. Physiological changes during pregnancy include an increase in circulating blood volume, increased resting heart rate, right ventricular dilatation, increase in pulmonary vascular resistance and a prothrombotic state, all of which can predispose to decompensation in patients with established pulmonary hypertension [5]. Late pregnancy, labor and the postpartum period are especially dangerous periods with rapid changes in systemic and pulmonary vascular resistance as well as intravascular blood volume. Established right heart failure in turn compromises placental oxygen transport and increases complication rates during the peripartum period [6]. Because of the known increased maternal and fetal mortality rates in patients with pulmonary hypertension [7, 8], current guidelines advise against planned pregnancies in such conditions and recommend termination of pregnancy if discovered early [6]. Despite this, there is growing experience with pregnancy in pulmonary hypertension patients including case reports describing management of acute decompensation of an established disease during the peripartum period [7–12]. In rare instances, previously undiagnosed pulmonary hypertension can become apparent during the late stage of pregnancy resulting in acute decompensation of right heart failure and resulting in a very difficult situation for the managing team. We present such a case and discuss important aspects of multidisciplinary management.

## Case presentation

A 34-year-old female at 30 weeks of gestation of her second pregnancy was admitted to the Department of Emergency Medicine at the Medical University of Pecs with worsening hypoxia, dyspnea, hemoptysis and oliguria. The patient had a history of hypothyroidism and thromboembolic events: she suffered a pulmonary embolism at age 20, shortly after initiation of an oral contraceptive medication. Subsequent work-up identified no hypercoagulability risk factors and oral anticoagulation therapy was discontinued after a year. She then suffered a DVT at age 31 after lower extremity fracture and was subsequently restarted on anticoagulation therapy, which was switched from oral to low molecular weight heparin during both her known pregnancies. Her previous pregnancy ended with intrauterine fetal demise 2 years prior at week 38. Her current pregnancy was unremarkable apart from occasional hemoptysis thought to be the side effect of anticoagulation therapy. Upon admittance, examination showed severe hypoxia (sO_2_ 69%), maintained blood pressure, sinus tachycardia, peripheral edema and diminished urine output. Echocardiography confirmed severe right heart dilatation and failure (RV cross section 50 mm, TAPSE 12 mm, systolic D sign, sPAP 77 + 15 = 92 mmHg), raising suspicion of acute pulmonary embolism (PE). Acute CT angiography scan was performed showing chronic pulmonary embolism in subsegmental arteries rather than acute PE. Lab findings were unremarkable. Obstetric assessment found a viable fetus. A critical care and PAH-specialist consult was requested to advise on thrombolysis, but based on the studies and presentation, acute decompensation of previously unknown pulmonary hypertension was diagnosed with no indication for thrombolysis. CPAP with high FiO_2_ was started and the patient was transferred to the Department of Anesthesiology and Intensive Therapy at Semmelweis University for further treatment.

**Fig. 1:**
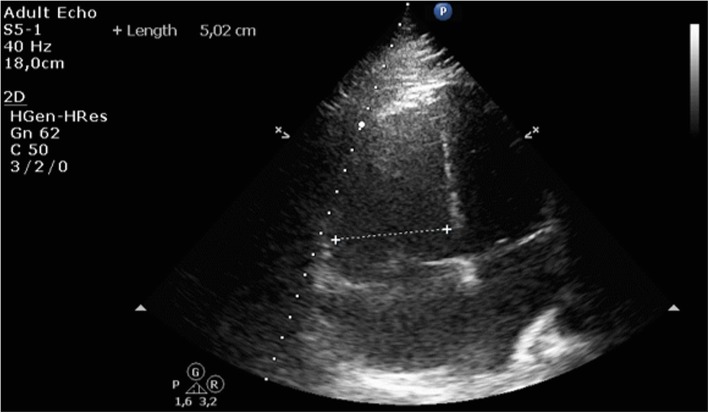
Apical 4-chamber view during echocardiography with right ventricle measurement

After the transfer, the patient was converted to BIPAP noninvasive ventilation (NIV) with high FiO_2_ to further improve oxygenation with a goal saturation of > 95%, central venous line and femoral PiCCO line as well as urinary catheter were placed. PiCCO showed diminished cardiac index (2.8 L/min/m^2^), while echocardiography continued to show diminished right ventricular function and severe dilatation (see Fig. 1). Lactate level was 3.5 mmol/L with a base excess (BE) of -8 mmol/L suggesting ongoing right heart failure with elevated pulmonary pressure.

Despite high FiO_2_ BIPAP NIV, we observed frequent transient desaturation episodes paired with severe dyspnea and concomitant episodes of hypotension suggestive of ongoing right heart failure. After multidisciplinary consult, a decision was made to stabilize the patient’s cardiovascular status and oxygenation, begin fetal pulmonary maturation treatment and schedule a Cesarean within the next 24 h. Cardiac anesthesia consultation was performed for possible acute ECMO requirement. To achieve preoperative cardiovascular stability, inotropic treatment was started (levosimendan in 0.07mcg/kg/min dose). Because of high observed pulmonary arterial pressures, clinical signs of right heart failure and expected possible worsening of hemodynamic parameters after surgery, a decision was made to start vasodilator treatment (intravenous epoprostenol, initially 2 ng/kg/min dose and quick titration to 7 ng/kg/min dose). A follow-up echocardiogram showed improvement (RV cross section 50 mm, TAPSE 12 mm, systolic D sign, sPAP 60 + 10 = 70 mmHg), while we observed improving lactate, BE levels and urine output with minimal diuretic therapy (20 mg furosemide every 12 h).

Anticoagulation (enoxaparin 60 mg SC used prophylactically during pregnancy) was discontinued for 12 h and after thromboelastography (TEG) evaluation of coagulation parameters to rule out prostacyclin-associated platelet dysfunction, an epidural catheter was placed. After improving lactate, BE and urine output, a Cesarean was performed under epidural anesthesia (300 mg lidocaine and 15mcg sufentanil) and continued noninvasive ventilation. Operating time was 35 min with a blood loss of 500 ml, levosimendan and epoprostenol was continued during the operation and cardiac output was monitored via PiCCO. Additional low dose norepinephrine (0.06mcg/kg/min) therapy was started to counteract anesthesia-induced decrease in systemic vascular resistance, maintaining stable blood pressure parameters in the perioperative period (preoperative BP: 124/62 mmHg, lowest intraoperative BP: 75/42 mmHg, postoperative BP: 118/62 mmHg). A 1490 g baby girl was delivered with an Apgar of 8/9. The baby required CPAP therapy and PICU treatment, but adapted as expected in subsequent days.

**Fig. 2:**
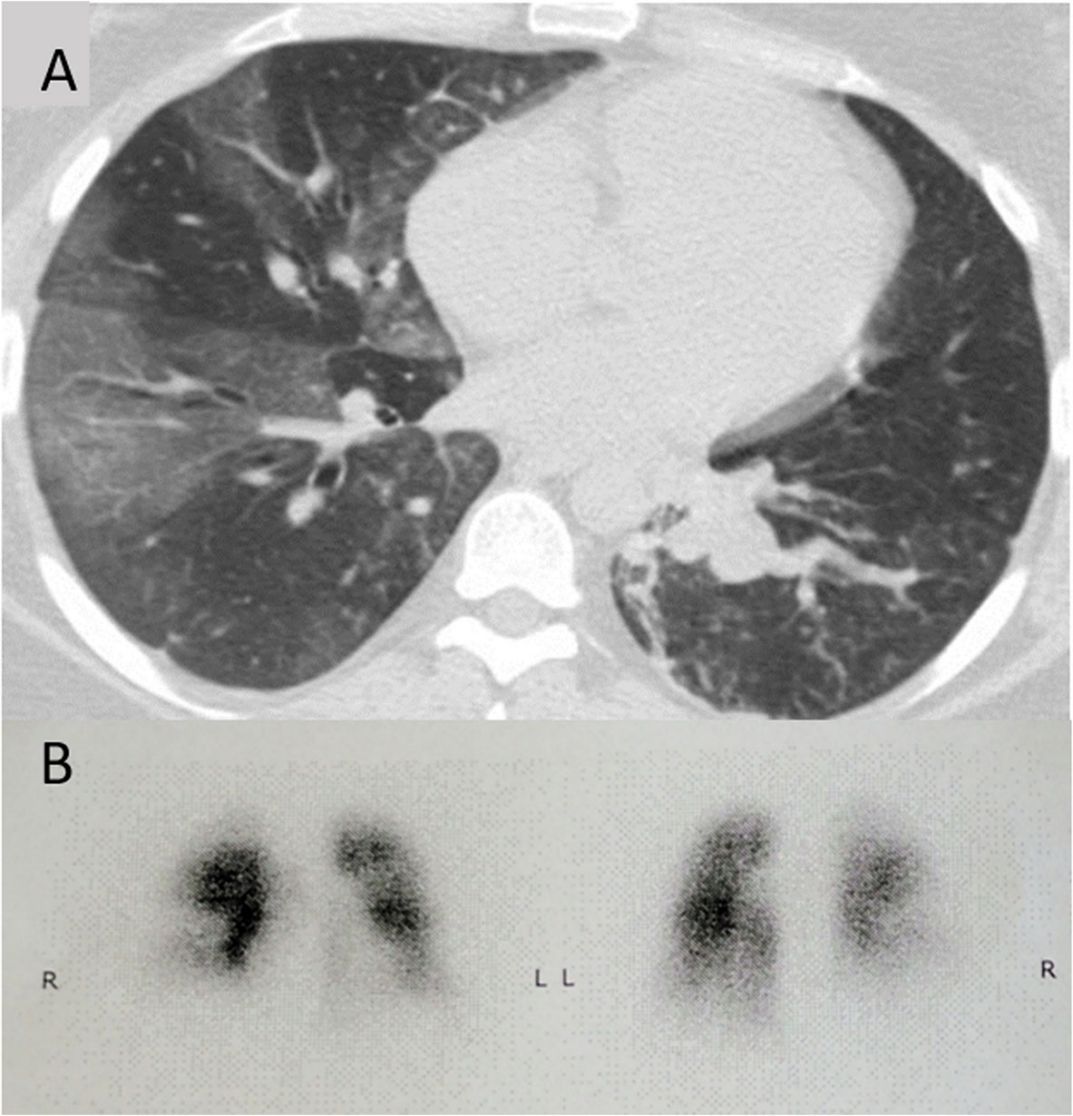
**a** Follow up chest CT scan showing obstruction of right lower lobe bronchi by blood clots as well as enlarged pulmonary vessels and mosaic pattern of lung parenchyma. **b** Anterior-posterior and posterior-anterior view of ventilation/perfusion scan showing bilateral perfusion deficits

The patient continued to be monitored during the postoperative period. Low molecular weight heparin anticoagulation was re-established 12 h after the operation and was regularly monitored with anti-Factor Xa activity. No bleeding complications were observed. Repeat echocardiogram showed improving right ventricle diameter (28 mm) but increased systolic pulmonary arterial pressure (72 + 10 = 82 mmHg). Epoprostenol dose was titrated up to 10 ng/kg/min, 24-h levosimendan treatment was followed with dobutamine treatment (4mcg/kg/min) and noninvasive ventilation was continued. During the next 48 h, we observed ongoing improvement in cardiac index (3.4 and subsequently 3.8 L/min/m^2^ by PiCCO), and in pulmonary pressure (sPAP 60 + 10 = 75 and subsequently 48 + 10 = 58 mmHg by echocardiogram). Urine output was maintained despite tapered diuretic therapy. Despite improving cardiovascular parameters, persistent BIPAP NIV and FiO_2_ dependence was observed, so chest CT was repeated with right lower lobe bronchi obstruction apparent (see Fig. 2). A bronchoscopy was performed in dexmedetomidine anesthesia and ongoing BIPAP noninvasive ventilation and several blood clots were extracted. After the procedure, oxygenation improved markedly and the patient was successfully weaned from BIPAP NIV to nasal O_2_ therapy.

Epoprostenol treatment was gradually converted to subcutaneous treprostinil therapy, treatment was escalated with digoxin, spironolactone and sildenafil, previously contraindicated for its possible systemic hypotension causing effect. Follow up echocardiography showed improving parameters. Right heart catheterization verified the diagnosis of pulmonary hypertension (PAP 78/35 mmHg, mPAP 49 mmHg, CO 5.33 L/min, PCWP 7 mmHg, PVR 7.88 Woods). Ventilation/perfusion scan was performed showing extensive segmental-subsegmental perfusion deficits in the right lung and scattered perfusion deficits in the left upper lobe (see Fig. 2), effectively establishing the diagnosis of chronic thromboembolic pulmonary hypertension with acute decompensation in the late stage of pregnancy.

Pulmonary endarterectomy was advised based on diagnosis and clinical symptoms, however given her continued hemodynamic stability, the patient’s preference was taken into consideration and the operation was postponed until optimal timing for mother and baby. The patient was discharged to the Pulmonology Department of the university after 15 days of ICU treatment. After further titration of treprostinil to 18 ng/kg/min, she was able to leave the hospital with her newborn after 4 weeks. Two- and 6-month follow-up showed further improvement without full resolution of echocardiography findings. She had a functional class (FC) of II (6-min walk test 570 m) with no observed side effects of subcutaneous PC treatment. Pulmonary endarterectomy is planned for a later date as per the patient’s wishes.

## Discussion and conclusions

Chronic thromboembolic pulmonary hypertension diagnosed during pregnancy carries considerable risk and requires adequate peripartum planning [12]. Most common symptoms include dyspnea, hemoptysis, exercise intolerance and careful workup can identify hypoxia and hypotension. If suspected early, BNP levels higher than 300 pg/mL can identify patients at risk for heart failure [13], while echocardiography can verify the existence of pulmonary hypertension and right ventricular involvement.

In some rare cases though, the condition remains unnoticed until sudden and progressive worsening of cardiorespiratory parameters. Understandably, the diagnosis and treatment of previously unknown pulmonary hypertension presenting as acute right heart failure and respiratory failure in the late stage of pregnancy poses a difficult multidisciplinary management problem. Increased maternal mortality, risk of fetal growth and developmental delay, high-risk delivery and potential worsening of circulatory parameters in the postpartum period, all need to be considered when treating the patient. Several issues need to be addressed when establishing a peripartum management plan for a patient newly diagnosed with acute decompensation of previously undiagnosed pulmonary hypertension (Table 1).

**Table 1:** Bullet points for peripartum management of acute decompensation of previously undiagnosed pulmonary hypertension

1.	Establish invasive hemodynamic monitoring
	• arterial, central line
	• consider pulmonary arterial catheter
	• consider PiCCO
2.	Provide respiratory support (aim for normoxia)
	• high flow O_2_
	• consider noninvasive ventilation
	• avoid invasive ventilation if possible
3.	Provide hemodynamic support (aim for normal cardiac output)
	• inotropic treatment: dobutamine, levosimendan
	• vasodilator treatment: intravenous prostacyclin
	• fluid management
4.	Assess ideal timing and mode of delivery
	• urgent/emergent Cesarean based on maternal and fetal assessment
5.	Choose ideal anesthetic technique
	• regional anesthesia if possible
6.	Continue postoperative monitoring
	• observe for increasing hemodynamic instability
	• observe for postpartum bleeding
7.	Assess ideal anticoagulation timing and agent
	• heparin vs. LMWH

As there are no current clear recommendations regarding these issues, the ideal management needs to be established on a case by case basis, which is highlighted by our experience. Perioperative invasive cardiovascular monitoring is critical, but pulmonary arterial catheter use is discouraged in acute right heart failure due to high complication rates [6]. Because of this, we opted to use PiCCO monitoring and regular echocardiography assessments. We hypothesized that regular measurement of cardiac index would be informative during the postpartum period when we expected hemodynamic instability. There are several factors that aid cardiovascular stability, crucial for both maternal and fetal outcome in the peripartum period. Improving oxygenation is important in reversing hypoxic pulmonary vasoconstriction, and normal oxygen saturation can ideally be achieved with high flow oxygen or low-pressure noninvasive ventilation. In our case we used BIPAP noninvasive ventilation with high FiO_2_: careful titration of pressures resulted in improved oxygenation while not compromising cardiovascular parameters.

Inotropic support can be achieved with dobutamine or levosimendan, which has recently been proven to be beneficial in acute right heart decompensation [14]. Vasopressors have the theoretical disadvantage of increasing pulmonary vascular resistance and should be used cautiously in patients with pulmonary hypertension. Choosing the optimal inotropes and vasopressors in a pregnant patient with pulmonary hypertension is further complicated by our limited knowledge on their effects on maternal and fetal circulation [15]. Dobutamine might reduce uterine blood flow and has a potent chronotropic effect [16]. Levosimendan compared to dobutamine has the potential benefits of decreasing short-term mortality in cases of acute heart failure [17] but has only been reportedly used in a handful of pregnant patients treated for cardiac shock or peripartum cardiomyopathy [18–20]. Although a previous publication noted the uterine relaxing effect of the drug [21], none of the aforementioned case reports cite postpartum bleeding as an adverse reaction. Norepinephrine as well as conventionally used second line vasopressors (dopamine, epinephrine) have been reported to reduce uterine blood flow, although norepinephrine seems to have no detrimental effect on the well-being of the fetus [22]. Ephedrine does increase uterine blood flow but has been associated with fetal acidosis [23]. Because of these facts, we opted to use an initial 24-h loading dose of levosimendan, followed by subsequent treatment with dobutamine started in the postpartum period and minimal dose norepinephrine to counteract systemic hypotension associated with anesthesia.

Severe decompensation of pulmonary hypertension also warrants vasodilator treatment [2], which in this situation is ideally achieved by rapidly titrated intravenous prostacyclin treatment. Additional vasodilator pharmacological options (endothelin receptor antagonists, phosphodiesterase type 5 inhibitors, guanylate cyclase stimulators) are generally limited because of possible fetal toxicity [6]. Use of mechanical cardiovascular support has been described in the peripartum period and despite increased bleeding risks and questionable long-term outcomes, it can be lifesaving during sudden cardiovascular collapse and therefore should be considered as an emergency intervention during delivery [7, 10, 11].

Although natural delivery has been described in well-managed cases with moderate pulmonary hypertension, delivery should be performed surgically in cases where cardiovascular stability is frail and exclusively in cases where right heart failure is established. Optimal timing for the surgery should be based on maternal and fetal evaluation. In our case, a Cesarean was warranted because of recurrent transient hypoxia, dyspnea and drops in maternal blood pressure. Ideally, optimizing cardiac output, oxygenation, acid-base parameters and urine output, as well as optimal anticoagulation timing, are crucial in order to minimize surgical risk. In our case, we scheduled the operation after a successful 24-h stabilization period, during which fetal lung maturation could also be initiated. Delivery should be performed with an aim of limited blood loss and short operation time (with gynecological and neonatal intensive care support). Choice of anesthesia for delivery is controversial. Use of general anesthesia has been described more frequently in literature although epidural anesthesia has the theoretical advantage of a slow build up and avoidance of positive pressure ventilation [9, 24]. As positive pressure ventilation is often associated with further sudden increase in pulmonary arterial pressure, we opted for slow buildup of epidural anesthesia, ongoing BIPAP noninvasive ventilation with high FiO_2_ and norepinephrine to counteract decrease of systemic vascular resistance.

The postpartum period is crucial, where patients should be vigilantly monitored for possible worsening of cardiovascular instability, post-partum complications and increased maternal mortality. Escalation of vasodilator and inotropic therapy might be required, as described in our case. Early initiation of anticoagulation therapy might be limited by postpartum and surgical bleeding, but ideally therapy should be started as soon as possible. Mechanical cardiovascular support and prostacyclin therapy are associated with increased bleeding complication rates [25]. We used low molecular weight heparin for anticoagulation guided by anti Xa Factor activity measurements.

Lastly, hemoptysis is a common presenting symptom of CTEPH [26] and can contribute to ongoing hypoxia by bronchial obstruction. This pathophysiology manifested in our described case where hypoxia and ventilator dependence continued despite improving hemodynamic parameters. If clinically relevant bronchial obstruction can be identified through imaging, bronchoscopic intervention is warranted. In our case, despite relevant bronchial obstruction and possible hemodynamic instability, invasive mechanical ventilation was successfully avoided during the procedure using dexmedetomidine sedation and ongoing noninvasive ventilation. The subsequent improvement in oxygenation might have contributed to improving hemodynamic stability. This case highlights the importance of adequate diagnosis, if ongoing hypoxia is present in these patients.

The case described illustrates that despite increased maternal and fetal risks, acute right heart decompensation discovered in the late stage of pregnancy can be managed successfully. Management should be attempted in an experienced cardiopulmonary critical care center with extracorporeal membrane oxygenation capability and advanced gynecological, obstetric anesthesiology and neonatal specialist teams. The key to successful treatment is stabilization of maternal cardiovascular parameters by optimizing oxygenation, inotropic support and vasodilator therapy. A Cesarean operation under epidural anesthesia performed urgently after stabilization is a viable option, while ongoing monitoring post-partum is required in the event that cardiovascular instability persists and mandates escalation of treatment.

## Data Availability

Not applicable

## Abbreviations

BE: Base excess
BIPAP: Bilevel positive airway pressure
BNP: Brain natriuretic peptide
BP: Blood pressure
CO: Cardiac output
CT: Computer tomography
CTEPH: Chronic thromboembolic pulmonary hypertension
DVT: Deep venous thrombosis
ECMO: Extracorporeal membrane oxygenation
FC: Functional class
FiO_2_: Fraction of inspired oxygen
IPAH: Idiopathic pulmonary arterial hypertension
LMWH: Low molecular weight heparin
mPAP: Mean pulmonary arterial pressure
NIV: Noninvasive ventilation
PAP: Pulmonary arterial pressure
PCWP: Pulmonary capillary wedge pressure
PE: Pulmonary embolism
PiCCO: Pulse contour cardiac output
PVR: Pulmonary vascular resistance
RV: Right ventricle
SC: Subcutaneous
sPAP: Systolic pulmonary arterial pressure
TAPSE: Tricuspid annular plane systolic excursion
